# Impact of Perioperative Massive Transfusion on Long Term Outcomes of Liver Transplantation: a Retrospective Cohort Study

**DOI:** 10.7150/ijms.61697

**Published:** 2021-10-15

**Authors:** Lingcan Tan, Xiaozhen Wei, Jianming Yue, Yaoxin Yang, Weiyi Zhang, Tao Zhu

**Affiliations:** Department of Anesthesiology, West China Hospital, Sichuan University & The Research Units of West China, Chinese Academy of Medical Sciences, No.37 Guoxue Street, Chengdu 610041, Sichuan Province, China.

**Keywords:** liver transplantation, massive transfusion, long-term outcomes, complications, risk factors

## Abstract

**Background:** Liver transplantation (LT) is associated with a significant risk of intraoperative hemorrhage and massive blood transfusion. However, there are few relevant reports addressing the long-term impacts of massive transfusion (MT) on liver transplantation recipients.

**Aim:** To assess the effects of MT on the short and long-term outcomes of adult liver transplantation recipients.

**Methods:** We included adult patients who underwent liver transplantation at West China Hospital from January 2011 to February 2015. MT was defined as red blood cell (RBC) transfusion of ≥10 units within 48 hours since the application of LT. Preoperative, intraoperative and postoperative information were collected for data analyzing. We used one-to-one propensity-matching to create pairs. Kaplan-Meier survival analysis was used to compare long-term outcomes of LT recipients between the MT and non-MT groups. Univariate and multivariate logistic regression analyses were performed to evaluate the risk factors associated with MT in LT.

**Results:** Finally, a total of 227 patients were included in our study. After propensity score matching, 59 patients were categorized into the MT and 59 patients in non-MT groups. Compared with the non-MT group, the MT group had a higher 30-day mortality (15.3% vs 0, p=0.006), and a higher incidence of postoperative complications, including postoperative pulmonary infection, abdominal hemorrhage, pleural effusion and severe acute kidney injury. Furthermore, MT group had prolonged postoperative ventilation support (42 vs 25 h, p=0.007) and prolonged durations of ICU (12.9 vs 9.5 d, p<0.001) stay. Multivariate COX regression indicated that massive transfusion (OR: 2.393, 95% CI: 1.164-4.923, p=0.018) and acute rejection (OR: 7.295, 95% CI: 2.108-25.246, p=0.02) were significant risk factors affecting long-term survivals of LT patients. The 1-year and 3-year survival rates patients in MT group were 82.5% and 67.3%, respectively, while those of non-MT group were 93.9% and 90.5%, respectively. The MT group exhibited a lower long-term survival rate than the non-MT group (HR: 2.393, 95% CI: 1.164-4.923, p<0.001). Finally, the multivariate logistic regression revealed that preoperative hemoglobin <118 g/L (OR: 5.062, 95% CI: 2.292-11.181, p<0.001) and intraoperative blood loss ≥1100 ml (OR: 3.212, 95% CI: 1.586-6.506, p = 0.001) were the independent risk factor of MT in patients undergoing LT.

**Conclusion:** Patients receiving MT in perioperative periods of LT had worse short-term and long-term outcomes than the non-MT patients. Massive transfusion and acute rejection were significant risk factors affecting long-term survivals of LT patients, and intraoperative blood loss of over 1100 ml was the independent risk factor of MT in patients undergoing LT. The results may offer valuable information on perioperative management in LT recipients who experience high risk of MT.

## Introduction

As an established intervention for patients with end-stage liver disease (ESLD), liver transplantation (LT) is associated with a significant risk of massive hemorrhage and massive blood transfusion 1,2. Owing to advances in surgical technique and anesthetic management, blood loss and blood product requirement has significantly decreased during LT over the past decade. Application of autologous blood transfusion during liver transplantation can greatly reduce allogeneic blood transfusion. In addition, low-dose tranexamic acid can improve the fibrinolytic state, prothrombin complex can improve coagulopathy, both can reduce intraoperative bleeding and blood consumption. Nevertheless, massive transfusion (MT) during LT still remains common. The cause of MT during OLT is multifactorial 3,4, the surgical reasons include major surgical hemorrhage, and hemostatic difficulty owing to lack of skilled surgical techniques, and the nonsurgical reasons are mainly coagulation dysfunction caused by coagulation factor deficiency, thrombocytopenia and hyperfibrinolysis.

Although administered to improve oxygen delivery to tissues or to restore hemostatic capacity, MT can lead to considerable adverse complications, such as profound coagulopathy, transfusion-related acute lung injury, acute kidney injury and infection 5. Consequently, MT is considered to be associated with early mortality and even worsened outcomes 6. Recent studies also have shown that MT may affect the recovery of liver function after transplantation and reduce the postoperative survival 7,8. However, to our knowledge, few studies have discussed the impacts of MT in perioperative periods of LT on long-term outcomes. Therefore, we conducted a retrospective cohort study to compare the short and long-term outcomes between adult LT recipients receiving MT and those not receiving MT.

## Materials and methods

### Study population

After obtaining approval from the Ethics Committee of West China Hospital of Sichuan University (protocol number 2016100), we analyzed data of LT recipients from January 2011 to February 2015 at the West China Hospital, Sichuan University, China. The inclusion criteria included adults with ESLD who received LT. Patients with fulminant hepatic failure patients, who received second LT, which received pre-operative RRT and pediatric LT recipients were all excluded. This retrospective cohort study compared the outcomes between patients who did and did not receive MT perioperative LT. MT was defined as red blood cell (RBC) transfusion of ≥10 units within 48 hours since the application of LT.

### Data collection

We collected the following preoperative information: patient demographic characteristics, indication of LT, abdominal surgical histories, presence of complications and comorbidities, Model for End-stage Liver Disease (MELD) Score and preoperative laboratory values including coagulation function, hemoglobin, platelet, leukocyte, creatinine and liver function. All diagnoses of primary diseases, comorbidities and complications were identified via the International Classification of Diseases (ICD-9) diagnostic codes. The KDIGO criteria used to define postoperative AKI (within 7 days after LT) were a 50% increase from the baseline (pre-operative value) serum creatine level or a 26.4 mmol/L increase from baseline within 48 h. Intra-operative data included type of LT, cold ischemia time, anhepatic phase, operating time, blood loss volume and fluid infusion volume. Types of LT included living donor liver transplantation or donation after circulatory death (DCD) liver transplantation. Post-operative data involved post-operative blood product requirements within 48 hours since LT, duration of mechanical ventilation, duration of ICU stay and hospitalization.

### Outcomes and follow-up

Our primary outcomes were all-cause 30-day mortality, 1-year survival rate and 3-year survival rate. Among of these, 30-day mortality was defined as short-term outcome, 1-year survival rate and 3-year survival rate were defined as long-term outcomes. All patients were followed from the index admission until 1 November 2020 or the date of death, whichever came first. The median follow-up period was 22.4 months (IQR, 2.3 to 72.3 months) for the MT group and 52.8 months (IQR, 13 to 65.9 months) for the non-MT group. Our secondary outcomes were in-hospital LT-related complications, including postoperative pulmonary infection, postoperative abdominal hemorrhage, pleural effusion, biliary leakage, anastomotic stenosis, arterial thrombosis, portal vein thrombosis, AKI stage II or III, acute rejection, ventilation support, ICU stay and hospital stay.

### Statistical analysis

We use statistical pack-age for the social sciences version 25.0 (SPSS Inc., Chicago, IL) to perform all statistics. Pearson's chi-square test was used to compare the categorical variables. Un-paired Student's t-test (presented as mean ± standard deviation) was used for normally distributed continuous variables. If normality was violated, the Mann-Whitney U test was used (presented as median with interquartile range).

To overcome selection bias and potential confounding factors, rigorous adjustments were performed using propensity scores matching. We matched each patient from the MT group with a counterpart from the non-MT group. The propensity score was the predicted probability to be in the MT group, derived from a given multivariable logistic regression value of covariates. The covariates in propensity score calculation included age, sex, BMI, abdominal surgical histories, preoperative comorbidities, MELD score, preoperative laboratory values and intraoperative data (type of LT, fluid infusion volume, cold ischemia time, anhepatic phase, operating time, blood loss volume). One-to-one optimal matches were assigned without replacement using a caliper of 0.15 maximum distances between propensity scores. Assessment of matching quality was performed comparing the two groups before and after PSM to check if significant differences in covariate statistics remained after the matching.

In order to identify risk factors affecting long-term prognosis and the risk factors predicting need of MT, all variables found to be significant upon univariable logistic regression were entered into multiple COX or logistic regression model. The p-value for inclusion in the multivariable regression was 0.1. The risk factors were assessed for its predictive ability to MT. The sensitivity, specificity and receiver operating characteristic (ROC) curve were determined to assess the predictive ability of MT risk factors. Survival curves were used to analyze long-term patient survival. For all tests, p value of <0.05 was considered statistically significant.

## Results

### Patients' characteristics

**Figure 1:**
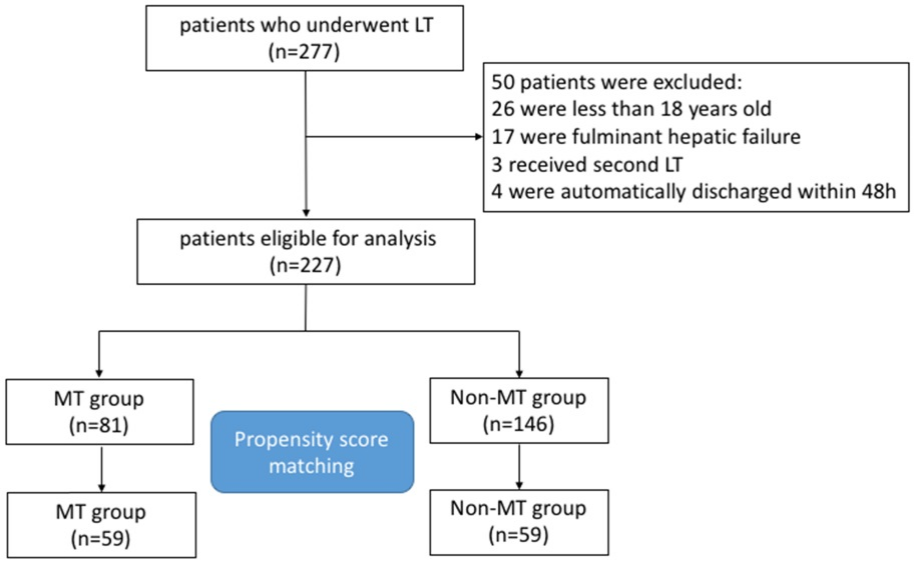
The flow chart of the study cohort.

We retrospectively reviewed medical records of 277 consecutive adult patients with ESLD who underwent LT from January 2011 to January 2015 at the West China Hospital, Sichuan University, China. Finally, 227 patients were eligible for inclusion in the present study. The study flow chart of the selection and exclusion criteria of patients is shown in Figure 1. For all patients, the major indications of LT application were hepatitis B or Hepatitis C associated hepatocellular carcinoma (HCC) (65.6%) and hepatitis B or Hepatitis C associated cirrhosis (30%), miscellaneous ESLD (4.4%) included primary or secondary cholestatic cirrhosis, alcoholic cirrhosis, autoimmune hepatitis, intrahepatic cholangiocellular carcinoma, and Wilson's disease (Table 1).

After matching, 59 patients were categorized into the MT and 59 patients in non-MT groups. The balance in factors between groups was assessed before and after matching. In the unmatched, significant differences between MT and non-MT group were observed in baseline characteristics, regarding hypertension and diabetes, MELD score, preoperative hemoglobin and platelet, preoperative coagulation function and liver function, as well as the volume of fluid infusion and blood loss, cold ischemia time, anhepatic phase and operating time (Table 1). However, after propensity matching, p-value > 0.05 for all baseline variables suggested an appropriate balance between the two groups (Table 2).

### Impacts of MT on postoperative complications

Table 3 also displays the short-term clinical outcomes in MT and non-MT patients. Compared with the non-MT group, the MT group had a higher 30-day mortality (15.3% vs 0, p=0.006). Compared with the non-MT group, MT group had higher incidences of some postoperative complications than non-MT group. Postoperative pulmonary infection was more common in MT group (45.8% vs 27.2%, p=0.035), and MT group experienced greater risks of abdominal hemorrhage (22% vs 3.4%, p=0.004) and pleural effusion (30.5% vs 10.2%, p=0.006). In addition, compared with non-MT group, MT group had a higher severe AKI (AKI stage II or III) incidence (18.6 % vs 1.7%, p=0.004). Furthermore, MT group had longer postoperative ventilation support (42 vs 25 h, p=0.007) and longer durations of ICU (12.9 vs 9.5 d, p<0.001) stay than the non-MT group. However, no significant difference was noted in durations of hospital stay between the two groups (27 d vs 26 d, p=0.393).

### Impacts of MT on long-term outcomes and risk factors affecting long-term outcomes

**Figure 2:**
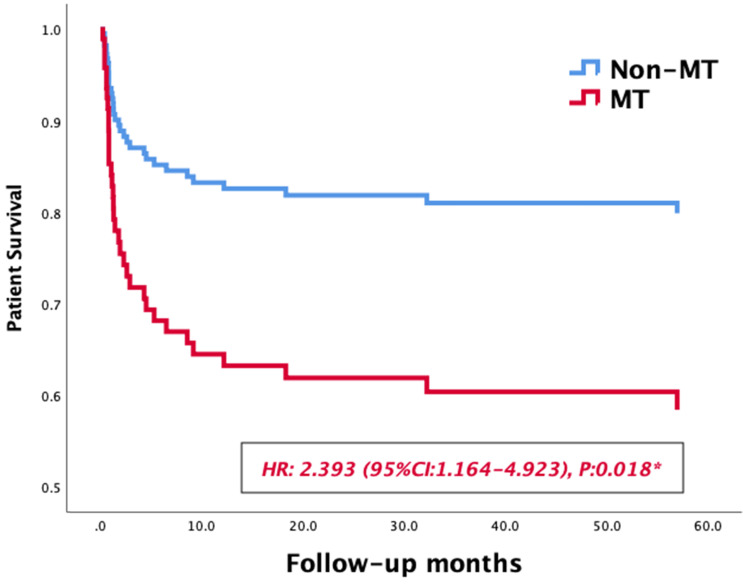
Survival curves of patients' survival in the MT and non-MT groups.

Massive transfusion, MELD Score, preoperative hemoglobin, intraoperative data and postoperative complications were selected for inclusion in the univariate Kaplan-Meier analysis. The medians of fluid infusion, blood loss, anhepatic phase, cold ischemia time and operating time after matching were set as the cutoff values of these variables, the results of univariate analysis are summarized in Table 4. Then a multiple COX regression model was performed to identify risk factors affecting long-term survivals of LT patients. As shown in Table 4, massive transfusion (OR: 2.393, 95% CI: 1.164-4.923, p=0.018) and acute rejection (OR: 7.295, 95% CI: 2.108-25.246, p=0.02) were significant risk factors affecting long-term survivals of LT patients. In survival curves, the 1-year and 3-year survival rate patients in MT group were 82.5% and 67.3%, respectively, while those of non-MT group were 93.9% and 90.5%, respectively. The MT group exhibited a lower long-term survival rate than the non-MT group (HR: 2.393, 95% CI: 1.164-4.923, p=0.018, Figure 2).

### Risk factors predicting the need for MT

**Figure 3:**
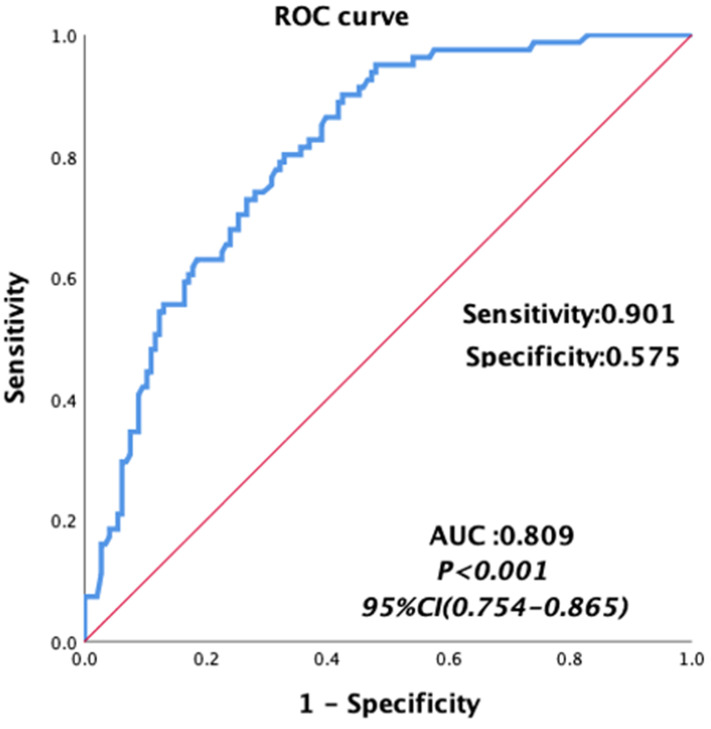
Area under the receiver operating characteristic curves for the model predicting MT in patients undergoing liver transplantation.

Univariate analyses were carried out for all preoperative and intraoperative factors that possibly affect MT. Among of these, the medians of preoperative continuous variables and intraoperative variables, before matching were set as the cutoff values of these variables. The univariate analysis showed that HCC, MELD score, albumin, total bilirubin, AST, hemoglobin, INR, PT, FIB and intraoperative blood loss, cold ischemia time and operation time were possibly related to MT, as shown in Table 5. Then we constructed a multivariable logistic regression analysis. In the multivariate logistic analysis, a forward stepwise elimination algorithm was used to retain risk factors in the final model when P = 0.05. Finally, the multivariate logistic regression analysis revealed that preoperative hemoglobin <118 g/L (OR: 5.062, 95% CI: 2.292-11.181, p<0.001) and intraoperative blood loss ≥1100 ml (OR: 3.212, 95% CI: 1.586-6.506, p = 0.001) were the independent risk factor of MT in patients undergoing LT (Table 5). The area under the receiver operating characteristic curve of the MT predictive model was 0.809, with a sensitivity of 0.901 and a specificity of 0.575 (95% CI: 0.754-0.865, p<0.001, Figure 3).

## Discussion

Numerous reports established a link between massive blood transfusion and serious complications, and may be associated with increased mortality. Nevertheless, limited information is available regarding the impact of MT on the long-term outcomes in LT patients. In this retrospective cohort study at our center, we aimed to study the impact of MT on the long-term outcomes in patients receiving LT. We found that MT patients had higher 30-day mortality (15.3% vs 0%) and higher incidence of postoperative complications than non-MT patients, such as postoperative pulmonary infection, postoperative abdominal hemorrhage, pleural effusion, AKI stage II or III. Meanwhile, MT patients experienced longer postoperative ventilation support and longer durations of ICU. Furthermore, the MT group exhibited a lower 1-year and 3-year survival rate than the non-MT group during the long term follow-up, indicating the significant adverse impacts of MT on both on short- and long-term clinical outcomes.

It is evident that the ability of concentrated red blood cells to adsorb vascular endothelial cells can be enhanced, and blood products can produce a large amount of inflammatory cytokines 9-11. Therefore, the infusion of excessive red blood cells can promote the systemic inflammatory response and induce immune activated lung injury 12. In addition, massive transfusion-associated circulatory overload can break the hydrostatic equilibrium and increase serous effusion 13-15. Besides, massive blood transfusion can lead to hypothermia and immunosuppression 16-18, thereby increasing the risk of short-term mortality, which is also confirmed by our study. On the other hand, massive blood transfusion may aggravate the body's iron load and induce redox reaction by catalyzing the production of oxygen free radicals, thus damage renal tubular epithelial cells and raise the risk of acute kidney injury 19-21. In contrast to earlier few findings 3,4, however, we found MT may have adverse impact on long-term prognosis in LT patients. A possible explanation for this might be that massive blood transfusion associated complications may affect the functional recovery of LT recipients, thereby worsen long-term outcomes. Thus, further research is necessary to investigate the possible mechanism of MT affecting the long-term outcomes of LT patients.

In the present study, we found that preoperative hemoglobin <118 g/L and intraoperative blood loss ≥1100 ml were the independent risk factors of MT in patients undergoing LT. Patients with ESLD are often accompanied by coagulation dysfunction and fibrinolysis dysfunction, which mainly due to the reduction of coagulation factors and platelet, the deficiency of platelet function and the hyperfibrinolysis 22-24. Several studies have concluded that preoperative anemia is associated with massive blood transfusion, our study confirmed these findings and further defined the degree of anemia 2,24. Preoperative coagulation disorders increase the risk of hemorrhage, then massive hemorrhage will aggravate existing anemia and increase the requirements of blood transfusion. Consistent with previous reports, our findings verified the predictivity of intraoperative blood loss on MT.

We infer that if intraoperative bleeding can be reduced, it may change the long-term prognosis of patients. What can be done to prevent intraoperative massive transfusion? Some new technology can be applied in liver transplantation to reduce blood loss. For example, maintaining the CVP low by restrictive intravenous fluid use and phlebotomy when tolerated may minimize portal congestion related bleeding 25. Studies have found that application of tranexamic acid combined with acute normovolemic hemodilution (ANH) can promptly supplement functional autologous platelets and coagulation factors, which can not only be used safely during surgery, but also reduce the transfusion of allogeneic blood 26, 27. In addition, thrombelastogram can help estimate coagulation function and guide the use of exogenous coagulation substances, make the correction of coagulation dysfunction more targeted and individualized, then may reduce the use of blood products 28. Furthermore, pulse-indicated continuous cardiac output (PICCO) monitoring and transesophageal echocardiography (TEE) can help guide the precise adjustment of intraoperative volume and avoid volume overload or even pulmonary edema 29, 30.

To our knowledge, the use of propensity match score to investigate and compare the impacts of MT in LT recipients has not been reported in previous publications. Similarities of matched groups in all aspects (preoperative variables and patient demographics) could well balance the confounding factors of retrospective cohort study, for maintaining baselines consistency in two groups and reducing selection bias. Unlike in previous studies 7, we excluded patients with fulminant hepatic failure, for these patients often already received massive blood transfusion before LT, and were often accompanied with severe comorbidities, such as gastrointestinal bleeding, severe infection, hepatorenal syndromes and requiring preoperative RRT, also with high MELD scores 31-34. We thought that severe pre-existing comorbidities might easily become uncontrollable confounding factor and interfere with the research results. After excluding a few special patients, final included study patients had relative low MELD scores, and there was a certain consistency between MELD and coagulation laboratory values. Maybe this can explain why preoperative coagulation did not predict MT, even univariate. On the other hand, the small sample size might also result in an inconsistency, and it requires further investigations.

Our study has some limitations. First, the features of the retrospective cohort study limits this study, and some data are missing. Second, our participant sample is modest in size and is drawn from a single institution. Hence, future multicenter researches should be carried on to attain larger cohorts in order to support or confute the findings of our study.

## Conclusions

Patients receiving MT in perioperative periods of LT had worse long-term outcomes than the non-MT patients. It was also noted that MT patients had higher risks of 30-day mortality. Moreover, the incidences of post-LT complications, such as pulmonary infection, abdominal hemorrhage, pleural effusion and severe acute kidney injury, were higher in the MT group. Massive transfusion and acute rejection were significant risk factors affecting long-term survivals of LT patients. Intraoperative blood loss of over 1100ml was the independent risk factor of MT in patients undergoing LT. The results may offer valuable information on perioperative management in LT recipients who experience high risk of MT.

## Figures and Tables

**Table 1:** Baseline characteristics of study patients before propensity score match

	Total (n=227)	MT (n=81)	Non-MT (n=146)	P-value
**Preoperative**				
Age (years)	46.2±9.7	46.9±9.7	45.8±9.6	0.877
Gender (male)	184 (81)	61 (75.3)	123 (84.2)	0.133
BMI	22.6 (20.3-25.0)	22.3 (19.7-25.0)	22.8 (20.8-25.0)	0.135
**Comorbidities**				
Hypertension	8 (3.5)	6 (7.4)	2 (1.4)	0.026*
Diabetes mellitus	19 (8.4)	12 (14.8)	7 (4.8)	0.012*
Abdominal surgical histories	143 (63)	54 (66.7)	89 (61)	0.473
MELD score	11 (9-17)	15 (10-20)	10 (8-14.5)	<0.001*
PT (s)	14.7 (12.8-17.4)	15.4 (13.6-19.4)	14.3 (12.6-15.7)	0.002*
APTT (s)	34.1 (30-42.9)	37.5 (30.6-48.2)	33.9 (29.7-40.2)	0.025*
TT (s)	21 (19.4-23.7)	21.4 (20-24.3)	20.9 (19.2-23.4)	0.061
FIB (g/L)	1.9 (1.4-2.7)	1.8 (1.2-2.3)	2.1 (1.5-2.9)	0.003*
INR	1.3 (1.1-1.5)	1.4 (1.2-1.7)	1.3 (1.1-1.4)	0.001*
Hemoglobin (g/L)	118 (93-141)	97 (82-119.5)	127 (105-145.3)	<0.001*
Platelet (10^9^/L)	73 (40-110)	63 (33-100.5)	76 (46-117.3)	0.016*
WBC (10^9^/L)	4.5 (3.1-6.2)	4.3 (2.8-6.6)	4.7 (3.2-6.1)	0.428
Total bilirubin (μmol/L)	26.7 (15.3-73.7)	54.7 (19.2-147.3)	22.9(13.9-42.7)	<0.001*
Creatinine (μmol/L)	71 (61.5-83.2)	71 (61.6-84)	71 (61.4-83.1)	0.742
Albumin (g/L)	35.7 (31-42)	32.6 (29.2-39.2)	37.9 (32.3-43.9)	<0.001*
AST (IU/L)	52 (36-87)	61 (41.5-106)	48 (32-81.5)	0.024*
ALT (IU/L)	39 (26-61)	40 (24.5-63)	39 (26-63)	0.824
**Indication of LT**				
HBV/HCV Cirrhosis	68 (30)	29 (35.8)	39 (26.7)	0.152
HBV/HCV HCC	149 (65.6)	47 (58)	102 (69.9)	0.072
Miscellaneous ESLD	10 (4.4)	5 (6.2)	5 (3.4)	0.334
**Type of Organ**				
Cadaveric donor	156 (68.7)	55 (67.9)	101 (69.2)	0.882
Live liver donor	71 (31.4)	26 (32.1)	45 (30.8)	0.876
Interoperative				
Fluid infusion (ml)	6500(5200-8000)	7000(5700-8700)	6450 (5000-7681)	0.02*
Cold ischemia time (h)	1.43 (1.15-1.78)	1.58 (1.24-2.04)	1.35 (1.12-1.73)	0.01*
Anhepatic phase (min)	85 (68-105)	91 (71-106.5)	80.5 (67-104)	0.19
Operating time (h)	8.8 (7.8-10.3)	9.3 (8.1-10.6)	8.6 (7.3-10)	0.021*
Blood loss (ml)	1100 (800-2000)	2000(1000-3750)	1000 (700-1500)	<0.001*

MT, massive transfusion; BMI, Body mass index; MELD, model for end-stage liver disease; PT, prothrombin time; APTT, activated partial thromboplastin time; TT, thrombin time; FIB, fibrinogen; INR, international normalized ratio; WBC, white blood cell; AST, aspartate aminotransferase; ALT, alanine aminotransferase, ESLD, end-stage liver disease.

**Table 2:** Baseline characteristics of study patients after propensity score match

	Total (n=118)	MT (n=59)	Non-MT (n=59)	P-value
**Preoperative**				
Age (years)	46.2±10.3	45.9±9.7	46.5±11.0	0.76
Gender (male)	93 (78.8)	45 (76.3)	48 (81.4)	0.653
BMI	22.0 (19.7-24.3)	21.5(19.2-23.9)	22.7 (20-24.4)	0.336
**Comorbidities**				
Hypertension	4 (3.4)	2 (3.4)	2 (3.4)	1
Diabetes mellitus	10 (8.5)	5 (8.5)	5 (8.5)	1
Abdominal surgical histories	72 (61)	36 (61)	36(61)	1
MELD score	12 (9-20)	13 (10-20)	12 (9-18)	0.525
PT (s)	15.3 (13.1-19.9)	15.5 (13.3-20)	15.1 (13.1-19.9)	0.764
APTT (s)	35.5 (31.6-46.4)	34.8 (29.8-49.1)	36.5 (32.4-43.3)	0.727
TT (s)	21.2 (20.0-24.0)	21.4 (20.2-24.4)	20.9 (19.6-23.1)	0.97
FIB (g/L)	1.9 (1.2-2.5)	1.8 (1.1-2.3)	1.9 (1.4-2.5)	0.478
INR	1.3 (1.2-1.7)	1.4 (1.2-1.7)	1.3 (1.1-1.8)	0.621
Hemoglobin (g/L)	104 (84-131)	98 (85-125)	107 (84-135)	0.685
Platelet (10^9^/L)	69 (34.8-103)	69 (34-113)	64 (37-94)	0.976
WBC (10^9^/L)	4.3 (2.7-6.3)	4.3 (2.7-6.6)	4.4 (2.7-6.3)	0.696
Total bilirubin (μmol/L)	31.2 (18.8-128.5)	34.1 (18.9-147.2)	29.3 (18.8-122.3)	0.434
Creatinine (μmol/L)	70.5 (62.0-83.0)	70.3 (62-83)	70.6 (62-81)	0.769
Albumin (g/L)	34.0 (30.5-39.9)	34.2 (29.9-40.4)	33.5 (30.8-39.2)	0.96
AST (IU/L)	52.5 (36.5-92.8)	56 (38-102)	49 (34-87)	0.776
ALT (IU/L)	36.5 (23.8-56.3)	40 (23-59)	36 (24-52)	0.914
Indication of LT				
HBV/HCV Cirrhosis	42 (35.6)	20 (33.9)	22(37.3)	0.848
HBV/HCV HCC	69 (58.5)	36(61)	33 (55.9)	0.709
Miscellaneous ESLD	7 (5.9)	3(5.1)	4 (6.8)	1
Type of Organ				
Cadaveric donor	81 (68.6)	41(69.5)	40 (67.8)	1
Live liver donor	37 (31.4)	18 (30.5)	19 (32.2)	1
Intraoperative				
Fluid volume (ml)	6300(5100-8000)	6500(5400-8200)	6100(5000-7800)	0.308
Cold ischemia time (h)	1.52 (1.15-1.86)	1.52 (1.17-1.83)	1.50 (1.12-1.95)	0.771
Anhepatic phase (min)	89 (69.25-107)	85 (70-105)	90 (67-115)	0.51
Operation time (h)	8.8 (7.9-10.2)	8.8 (8.0-11.7)	8.7 (7.8-10.6)	0.848
Blood loss (ml)	1500(1000-2500)	1500(1000-3000)	1400 (900-2000)	0.138

MT, massive transfusion; BMI, Body mass index; MELD, model for end-stage liver disease; PT, prothrombin time; APTT, activated partial thromboplastin time; TT, thrombin time; FIB, fibrinogen; INR, international normalized ratio; WBC, white blood cell; AST, aspartate aminotransferase; ALT, alanine aminotransferase; ESLD, end-stage liver disease.

**Table 3:** In-hospital complications and complications in the propensity score matched cohort

	Total (n=118)	MT (n=59)	Non-MT (n=59)	P-value
30-day mortality	9 (7.6)	9 (15.3)	0(0.0)	0.006*
Pulmonary infection	43 (36.4)	27 (45.8)	16 (27.2)	0.035*
Abdominal hemorrhage	15 (12.7)	13 (22)	2 (3.4)	0.004*
Pleural effusion	24 (20.3)	18 (30.5)	6 (10.2)	0.006*
Biliary leakage	2 (1.7)	1 (1.7)	1 (1.7)	1.0
Anastomotic stenosis	9 (7.6)	6 (10.2)	3 (5.1)	0.49
Arterial thrombosis	3 (2.5)	2 (3.4)	1 (1.7)	1.0
Portal vein thrombosis	6 (5.1)	4 (6.8)	2 (3.4)	0.67
AKI stage II or III	12 (10.2)	11 (18.6)	1 (1.7)	0.004*
Acute rejection	4 (3.4)	2(3.4)	2 (3.4)	1.0
Ventilation support (h)	31 (12-62)	42 (14-119)	25 (11-37)	0.007*
ICU stay (d)	10.8 (7.9-16.6)	12.9 (9.8-18.5)	9.5 (7.5-12.3)	<0.001*
Hospital stay (d)	27 (19-42)	27 (19-45)	26 (20-38)	0.393

MT, massive transfusion; AKI, acute kidney injury; ICU, intensive care unit.

**Table 4:** Risk factors affecting long-term outcome in LT patients

Variables	Categorization	Univariate Analysis	Multivariable Analysis	
		P-value	P-value	OR (95% CI)
Massive transfusion	absent, present	0.03*	0.018*	2.393 (1.164-4.923)
MELD Score	<12, >12	0.445		
Hemoglobin (g/L)	<104, ≥104	0.682		
Fluid infusion (ml)	<6300, ≥6300	0.751		
Blood loss (ml)	<1500, ≥1500	0.251		
Anhepatic phase (min)	<89, ≥89	0.313		
Clod ischemia time (h)	<1.52, ≥1.52	0.914		
Operating time (h)	<8.8, ≥8.8	0.539		
Pulmonary infection	absent, present	0.103		
Pleural effusion	absent, present	0.324		
Abdominal hemorrhage	absent, present	0.032*		
Acute rejection	absent, present	0.002*	0.02*	7.295 (2.108-25.246)
Anastomotic stenosis	absent, present	0.725		
Biliary leakage	absent, present	0.435		
Arterial thrombosis	absent, present	0.726		
Portal vein thrombosis	absent, present	0.858		
AKI stage II or III	absent, present	0.121		

MELD, model for end-stage liver disease; AKI, acute kidney injury.

**Table 5:** Risk factors for massive transfusion in all LT patients (n=227)

Variables	Categorization	Univariate Analysis		Multivariable Analysis	
		OR (95% CI)	P-value	OR (95% CI)	P-value
**Preoperative**					
Cirrhosis	Negative, positive	1.53 (0.854-2.74)	0.153		
HCC	Negative, positive	0.937 (0.873-1.006)	0.073		
Abdominal surgical histories	Absent, present	1.281 (0.725-2.263)	0.394		
MELD Score	<11, ≥11	2.664 (1.489-4.767)	0.001*		
Albumin (g/L)	<35.7, ≥35.7	2.57 (1.466-4.505)	0.001*		
Total bilirubin (μmol/L)	<26.7, ≥26.7	2.429 (1.387-4.225)	0.002*		
AST (IU/L)	<52, ≥52	1.668 (0.963-2.891)	0.068		
ALT (IU/L)	<39, ≥39	0.997 (0.579-1.717)	0.992		
Hemoglobin (g/L)	<118, ≥118	5.419 (2.83-9.355)	<0.001*	5.062 (2.292-11.181)	<0.001*
Platelet (10^9^/L)	<73, ≥73	1.55 (0.897-2.678)	0.117		
INR	<1.3, ≥1.3	1.916 (1.104-3.325)	0.021*		
PT (s)	<14.7, ≥14.7	2.067 (1.187-3.598)	0.01*		
APTT (s)	<34.1s , ≥34.3s	1.715 (0.99-2.972)	0.055		
FIB (g/L)	<1.9, ≥1.9	2.052 (1.183-3.561)	0.001*		
**Intraoperative**					
Blood loss, ml	<1100, ≥1100	4.727 (2.597-8.607)	<0.001*	3.212 (1.586-6.506)	0.001*
Anhepatic phase, mins	<85, ≥85	1.668 (0.963-2.891)	0.068		
Clod ischemia time, hours	<1.43, ≥1.43	1.763 (1.017-3.056)	0.043*		
Fluid volume, ml	<6500, ≥6500	1.715 (0.99-2.972)	0.055		
Operating time, hours	<8.8, ≥8.8	1.955 (1.123-3.402)	0.018*		

MT, massive transfusion; hepatocellular carcinoma (HCC); MELD, model for end-stage liver disease; PT, prothrombin time; APTT, activated partial thromboplastin time; TT, thrombin time; FIB, fibrinogen; INR, international normalized ratio.

## Abbreviations

BMI: Body mass index
LT: liver transplantation
MT: massive transfusion
RBC: red blood cell
ESLD: end-stage liver disease
MELD: Model for End-stage Liver Disease
HBV: hepatitis B virus
HCV: hepatitis C virus
DCD: donation after circulatory death
AKI: acute kidney injury
MT: massive transfusion
HCC: hepatocellular carcinoma
INR: international normalized ratio
APTT: activated partial thromboplastin time
PT: prothrombin time
TT: thrombin time
FIB: fibrinogen
Hb: hemoglobin
PLT: Platelet
WBC: white blood cell
AFP: alpha-fetoprotein
AST: aspartate aminotransferase
ALT: alanine aminotransferase
